# Understanding histotripsy ablation safety: a systematic review of pre-clinical and clinical adverse events

**DOI:** 10.1007/s44343-026-00062-2

**Published:** 2026-10-12

**Authors:** Madeline I. Foo, Cynthia Uzoukwu, Robert Morhard, Katrina L. Falk, Bradford J. Wood, Gregory Frey, Timothy J. Ziemlewicz, Eli Vlaisavljevich, Beau B. Toskich, Alissa Hendricks-Wenger

**Affiliations:** 1 Department of General Surgery, Johns Hopkins Hospital University, Baltimore, MD USA; 2 Department of Interventional Radiology, Johns Hopkins Hospital University, Baltimore, MD USA; 3 Northeastern Ohio Medical University, Rootstown, OH USA; 4 Center for Interventional Oncology, Radiology and Imaging Sciences, Clinical Center, National Institutes of Health, Bethesda, MD USA; 5 Department of Radiology, University of Wisconsin School of Medicine and Public Health, Madison, WI USA; 6 Department of Oncology and Department of Radiology, University of Milan, Milan, Italy; 7 DeepSight Technology Inc, Santa Clara, CA USA; 8 Division of Interventional Radiology, Mayo Clinic Florida, Jacksonville, FL USA; 9 Department of Biomedical Engineering & Mechanics, Virginia Tech, Blacksburg, VA USA

**Keywords:** Histotripsy, Ablation, Safety, Complications, Adverse events, Focused ultrasound

## Abstract

Histotripsy is the first cavitation-based ablation modality. Given limited clinical experience, synthesis of pre-clinical and early clinical data is essential to guide safe adoption. A PubMed and SCOPUS search was performed focused on pre-clinical and clinical adverse events (AEs) from Histotripsy ablation of parenchymal tissues in vivo (*n* = 141 total studies, *n* = 27 clinical reports). Early data suggest an acceptable acute safety profile in select settings; however, reports of serious AEs including pre-clinical bowel perforations, clinical acute kidney injuries, clinical vascular injuries and thromboses, and a clinical case report of hyperprogression raise caution. These highlight the need for proper technique, understanding of mechanism nuances, and longer-term follow-up to assess effects. Recognition of mechanism-specific AEs provides a framework to guide future assessments and safer adoption of Histotripsy.

**Level of Evidence** No level of evidence (review).

## Introduction

**Fig. 1:**
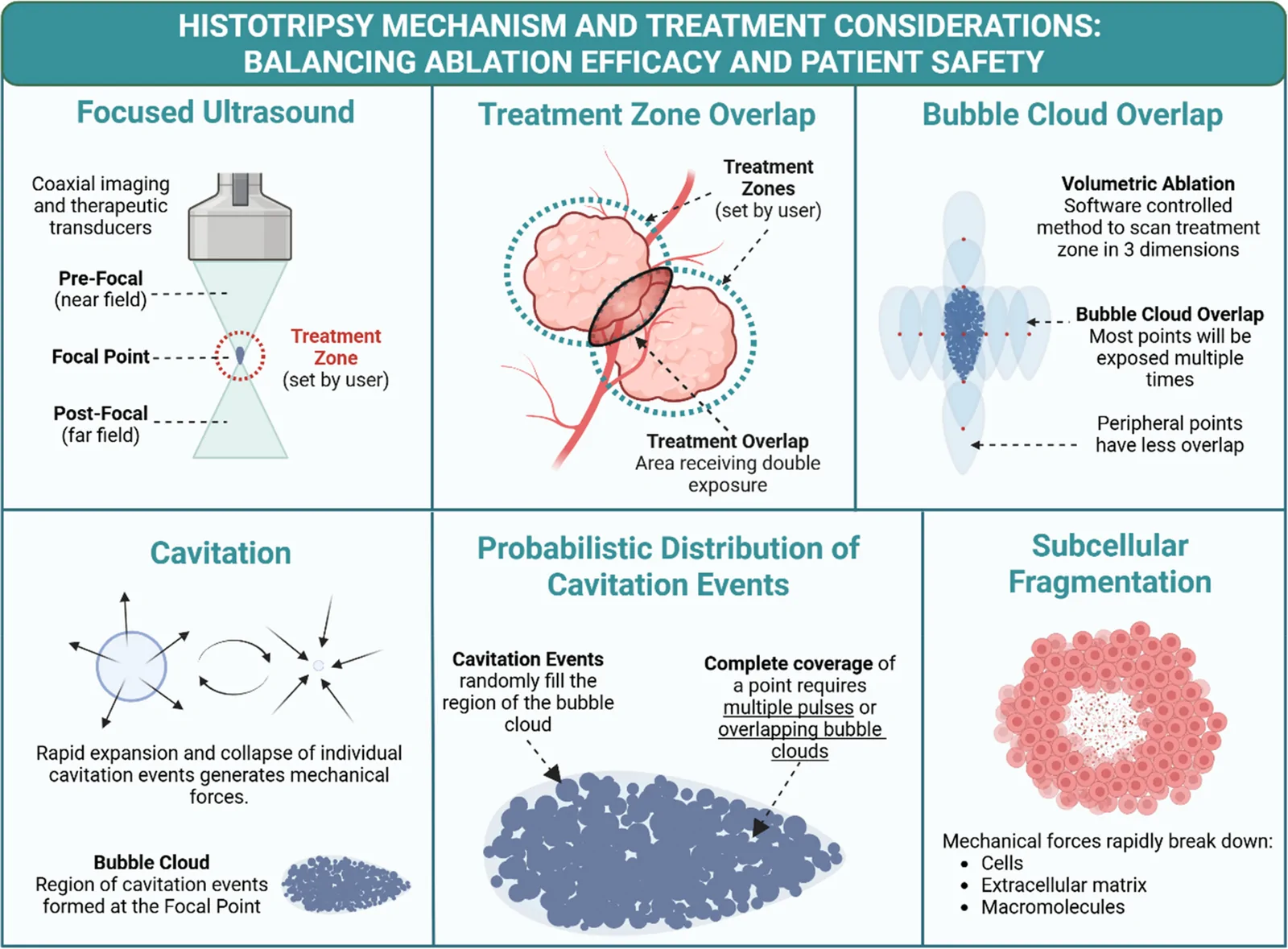
Overview of histotripsy mechanisms and treatment considerations. Created in BioRender

Histotripsy is a focused-ultrasound ablation modality that generates focal cavitation to mechanically fractionate tissues, covering a user-determined volumetric treatment zone with an automated scanning protocol (Fig. 1) [24, 50, 106, 115]. Several potential adverse events (AEs) follow from this mechanism. Injury within the ablation zone is common to all ablation modalities, and has thus been a focus of pre-clinical studies [41, 80, 95, 97, 108]. Pre-focal cavitation and thermal effects can occur at tissue-tissue interfaces without appropriate mitigation [65, 67]. Post-focal injury has a theoretical risk for highly susceptible tissues, especially gas-filled tissues.

Rapid liquefaction bypasses conventional thermal cell death mechanisms, releasing intracellular electrolytes and macromolecules [27], which may contribute to local and systemic injury [15]. Given that cavitation events occur with a probabilistic distribution, adequate-appearing treatments may leave viable cells [28], create debris that could embolize vasculature [114], and theoretically contribute to metastatic spread [7].

This systematic review evaluates animal and human evidence on histotripsy-associated AEs and highlights gaps in the literature to guide further mechanistic studies and post-market assessment supporting safe clinical adoption.

## Methods

### Search strategy and study identification

A search of PubMed and SCOPUS using the keyword “Histotripsy” on June 22, 2026 identified *n* = 1270 potential publications. Inclusion criteria: (1) used Histotripsy for in vivo ablation, (2) targeted parenchymal tissue, (3) peer-reviewed scientific publications, (4) full-text available in English. Exclusion criteria: (1) conference abstracts, reviews, preprints, (2) articles without results, (3) unpublished articles/theses, (4) only targeted non-parenchymal tissues, including: gelatin, phantoms, abscesses, and thromboses.

Studies were included regardless of Histotripsy subtype (shock scattering, boiling, cavitation-cloud) or tissue targeted; subtypes were not differentiated. Other focused-ultrasound modalities were excluded to restrict analysis to non-thermal cavitation mechanisms.

**Fig. 2:**
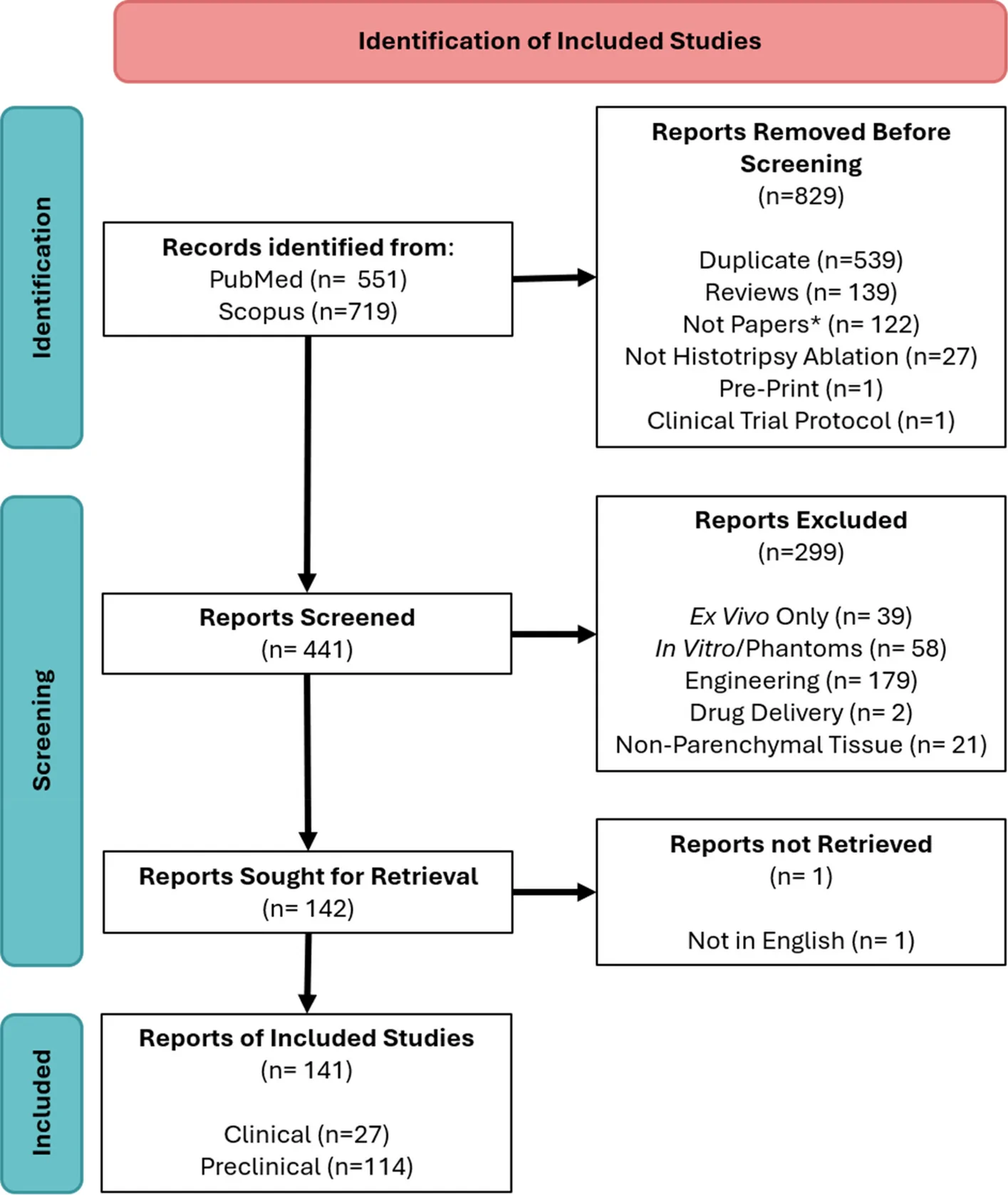
PRISMA Flow Diagram of Report Identification, Screening, and Inclusion Process. The asterisk (*) indicating the category ‘Not Papers’ includes abstracts, news articles, conference proceedings, theses, and other similar sources

Full-text evaluation manually identified studies using Histotripsy for in vivo parenchymal tissue ablation (*n* = 141). Process completed and verified by multiple authors and is summarized in Fig. 2.

### Extraction and evaluation of adverse event data

Full-text articles were reviewed with data systematically documented. Formal meta-analysis was only conducted on clinical cohort studies of hepatic ablation; remaining findings were qualitatively synthesized. The severity of AEs was classified according to the Society of Interventional Radiology Adverse Events (SIR-AE) severity classification [11]. Process completed and verified by multiple authors.

Non-target tissues were defined as anatomy not intended for ablation. Location categorized as (1) prefocal, between the transducer and the focus, (2) post-focal, deep to the treatment zone along the ultrasound path, or (3) within the prescribed treatment volume. Studies were reviewed for incidences of bleeding. When available, thrombus or embolus size, distribution, and clinical consequences were recorded. Studies were evaluated for reports of acute metastases or acute tumor seeding following Histotripsy treatment.

## Results

### Characteristics of included studies

There were 114 animal and 27 human studies (Table 1). Most animal studies were on healthy tissues (62/114) and used murine or porcine models (33/114 and 35/114, respectively). Clinical studies focused on hepatic malignancies (25/27) with only 5/27 studies reporting more than 6 months of follow-up. Animal studies frequently confirmed ablation with histopathology or histopathology and imaging (32/114 and 67/114, respectively); only 2/27 clinical studies referenced histopathology [7, 90].

**Table 1:** Study characteristics. Characteristics included Histotripsy studies, reported separately for pre-clinical and clinical evidence. Values are *n* (%) unless otherwise stated. Percentages are based on column-specific denominators

Characteristic	Pre-clinical (%)	Clinical (%)
Studies	114	27
**Timeline**		
First publication year	2006	2018 (prostate); 2021 (liver)
Years of published evidence	20	1 (prostate); 5 (liver)
**Animal model**		
Mouse	33 (28.9%)	—
Rat	14 (12.3%)	—
Rabbit	6 (5.3%)	—
Pig	35 (30.7%)	—
Dog	22 (19.3%)	—
Other	4 (3.5%)	—
**Tumor model**		
Healthy or non-malignant tissue	62 (54.4%)	1 (3.7%)
Subcutaneous tumor	23 (20.2%)	—
Orthotopic tumor	21 (18.4%)	—
Spontaneous tumor	8 (7.0%)	26 (96.3%)
**Target organ**		
Liver	26 (22.8%)	25 (92.6%)
Kidney	12 (10.5%)	1 (3.7%)
Liver + Kidney	5 (4.4%)	—
Prostate/periprostatic structures	12 (10.5%)	1 (3.7%)
Pancreas	4 (3.5%)	—
Flank/Subcutaneous	21 (18.4%)	—
Breast	3 (2.6%)	—
Appendicular	2 (1.8%)	—
Brain	10 (8.8%)	—
Bone	8 (7.0%)	—
Heart	4 (3.5%)	—
Other	7 (6.1%)	—
**Follow-up**		
Acute only	40 (35.1%)	0 (0.0%)
≤ 7 days	24 (21.1%)	0 (0.0%)
> 7 days	31 (27.2%)	8 (29.6%)
> 1 month	19 (16.7%)	14 (51.9%)
> 6 months	0 (0.0%)	5 (18.5%)
**Assessment method**		
Histopathology only	32 (28.1%)	0 (0.0%)
Imaging only	9 (7.9%)	25 (92.6%)
Histopathology and imaging	67 (58.8%)	2 (7.4%)
Neither (other assays only)	6 (5.3%)	0 (0.0%)

Follow-up rows are mutually exclusive tiers by maximum follow-up: > 7 days = 8–30 days; > 1 month = 31–180 days; > 6 months = > 180 days

Assessment-method rows are mutually exclusive. “Neither” = studies assessed only by flow cytometry/molecular/serum-biomarker assays (no tissue histology, no imaging)

Clinical study characteristics are detailed in Table 2. The THERESA trial included an additional case report [92, 93], and the #HOPE4LIVER has been reported twice [50, 116]. Assuming no additional overlaps in reporting, there could be a maximum of 607 unique patients. Pre-clinical studies were not broken down given inconsistent reports.

**Table 2:** Summary of clinical study characteristics

**Reference**	**Country**	**Center**	**Cohort overlap**	**Primary objective**	**Patients** (N)	**Lesions** (N)		**Follow-up**	**Target organ**
**Registered, multicenter, prospective, single arm, cohort study**									
Schuster, 2018 [75]	USA	Multicenter (2)	None	Safety and Technical Success	25	25	6 mo		Prostate
Vidal-Jove, 2022 [93]	Spain	Multicenter (2)	Yes (A)	Safety and Technical Success	8	11	2 mo		Liver
Mendiratta-Lala, 2024 [50]	USA, Spain, Germany, UK, Italy	Multicenter (14)	Yes (B)	Safety and Technical Success	44	49	1 mo		Liver
Ziemlewicz, 2025 [116]	USA, Spain, Germany, UK, Italy	Multicenter (14)	Yes (B)	Outcomes	47	52	12 mo		Liver
**Multicenter, retrospective, single arm, cohort study**									
Wehrle, 2025 [100]	USA	Multicenter (4)	Possible	Outcomes	47	91	1 mo		Liver
Wehrle, 2025 [101]	USA	Multicenter (9)	Possible	Safety	230	295	1 mo		Liver
**Multicenter case series**									
Poe, 2026 [62]	USA	Multicenter (5)	Yes (C)*	AE Report	9	12	1 mo		Liver
Breuer, 2026 [9]	USA	Multicenter (4)	Yes (C)*	AE Report	4	6	< 1 mo		Liver
**Prospective, single arm, cohort study**									
Wehrle, 2026 [99]	USA	Single	Possible	Outcomes	71	109	3 mo		Liver
Chan, 2026 [12]	China	Single	None	Outcomes	30	60	1 mo		Liver
**Single center, retrospective, single arm, cohort study**									
Mabud, 2025 [47]	USA	Single	Possible	Outcomes	26	56	3 mo		Liver
Jimenez-Soto, 2026 [31]	USA	Single	None	Outcomes	27	42	12 mo		Liver
Mabud, 2025 [46]	USA	Single	Possible	Outcomes	21	39	6 mo		Liver
Liu, 2026 [44]	USA	Single	None	Outcomes	27	66**	NR		Liver
**Single center case series**									
Worlikar, 2026 [108]	USA	Single	Possible	AE Management	21	21	1 mo		Liver
Cook, 2026 [13]	USA	Single	None	AE Report	3	5	4 mo		Liver
**Case report**									
Wong, 2025 [107]	USA	Single	None	AE Report	2	3	1 mo		Liver
Berman, 2025 [7]	USA	Single	None	AE Report	1	1	3 mo		Liver
Bhutta, 2026 [8]	USA	Single	None	AE Report	1	1	< 1 mo		Liver
Wah, 2025 [98]	UK	Single	None	Novel Application	1	NR	6 mo		Kidney
Burns, 2025 [10]	USA	Single	Possible	Novel Application	1	NR	8 mo		Liver
Ong, 2026 [57]	USA	Single	Possible	Novel Application	1	NR	6 mo		Liver
Uysal, 2025 [90]	USA	Single	Possible	Novel Application	1	1	1 mo		Liver
Juarez, 2026 [32]	USA	Single	Possible	Novel Application	1	10	7 mo		Liver
Vidal-Jové, 2021 [92]	USA	Single	Yes (A)	Systemic Response	1	1	12 mo		Liver
Ong, 2026 [56]	USA	Single	Possible	Systemic Response	1	NR	5 mo		Liver
Staley, 2026 [82]	USA	Single	None	Systemic Response	1	1	7 mo		Liver

Cohort Overlap: Grouped when specific, reported as ‘Possible’ when there is overlap in treatment centers and timeframe. Follow Up: Longest follow-up reported in the study, rounded to the nearest month (mo)

^*^Report confirmed overlap of 2 treated patients

^**^Exact lesion number not specified, number in table based on minimum number plausible

Pre-clinical platforms frequently utilized customized devices, based on mechanical properties and acoustic considerations, while clinical studies have used either the VORTX Rx or Edison systems (Table 3). Pre-clinical studies routinely specified therapeutic ultrasound parameters including the Histotripsy mechanism, transducer frequency, and a measure of acoustic delivery, comparable to the clinical standardized reporting criteria for ablation [2]. Clinical studies did not include comparable data, with no clear reporting of the Edison system’s acoustic parameters.

**Table 3:** Device parameters reported in studies using histotripsy for parenchymal tissue ablation

Platform	Studies	Histotripsy mechanism(s)	Therapeutic transducer frequency	Reported measurements of acoustic delivery
**Pre-clinical systems**	Most pre-clinical studies Veterinary clinical trials	Intrinsic-threshold histotripsy Shock-scattering histotripsy Boiling histotripsy	250 kHz–3 MHz	Pulse Repetition Frequency (PRF) Cloud Overlap or Spacing Pulses Per Location (PPL) Peak Negative Pressure
**VORTX Rx**	BPH Trial [75] THERESA [92, 93]	NR	700 kHz	NR
**Edison**	#HOPE4LIVER [50, 116] All other clinical reports Pre-clinical studies	NR^*^	NR	Volume Treated (per lesion) Overlapping Treatments (Binary) Relative Voltage (% unspecified limit)

NR = not reported in key clinical peer-reviewed publications

^*^One clinical case report states intrinsic-threshold histotripsy [82], and another reported the transducer to have 52-elements[107]

### Summary of adverse events

Reported AEs are summarized in Table 4. Of note, gastrointestinal and pulmonary AEs were only reported pre-clinically, while vascular injuries, acute kidney injuries (AKIs), and disease progression were only reported clinically.

**Table 4:** Histotripsy adverse events summarized by category. Adverse events are grouped by injury class. The table distinguishes true adverse events from intended treatment-zone effects and imaging-only, self-resolving findings. Counts are reported per study and are not pooled; studies are referenced as appropriate

	**Pre-clinical evidence**	**Clinical evidence**	**Proposed mechanism**
**General local & off-target injuries**			
Gastrointestinal	• Bowel perforation when treatment zone intentionally included small bowel [26] • Transmural (*n* = 1/12) and partial-thickness (*n* = 11/12) gastric injury when treatment zone intentionally include stomach [38] • Septic peritonitis and GI obstruction after off-target ablation during porcine pancreatic ablation [21] • Pre-focal intestinal bruising from bowel gas during porcine pancreas ablation [20]	• No clinical reports	Direct cavitation when treatment margin includes bowel or stomach [26, 38], secondary to poor ultrasound visualization [21], and pre-focal cavitation seeded by bowel gas [20]
Pancreatitis	• Pancreatitis (*n* = 2) after histotripsy of healthy porcine pancreas [21]	• Mild interstitial pancreatitis (*n* = 1) on 12-h MRI (transient amylase rise to 244 U/L, asymptomatic) [107]	Direct cavitation when treatment zone intentionally includes pancreas [21] or water-bath compression pushes pancreas into treatment zone [107]
Skin and soft-tissue	• Warmth, erythema, edema, petechiae or minor injury across studies [19, 37, 40, 42, 65, 68, 69] • Post-focal skin injury (*n* = 4/24) including SIR-AE 3 ulcerations (*n* = 2/24) in limb-sparing osteosarcoma treatment [91]	• Skin blistering (SIR-AE 1, *n* = 1) [46] • Wrist rash (*n* = 1) [31]	Pre-focal cavitation at the skin surface [65], post-focal thermal accumulation in distal limbs [91], and non-treatment pressure from the coupling water bath [68]
Pulmonary	• Pulmonary hemorrhage with alveolar septal rupture in porcine and murine models [40, 103]	• No clinical reports	Pre-focal injury to aerated lung [40]
Urinary tract	• Urethral injuries and leaks during prostate treatments [37, 71, 73, 74, 84], protected at lower doses [23, 43] • Rectoprostatic fistulas in canine prostate treatment [73] • Ureteral debris causing collecting-system clots [33, 37, 41, 71, 73, 74, 84]	• Mild transient hematuria after renal [98] and prostatic [75] treatments	Direct cavitation when treatment margin intentionally includes urinary structures, mitigated by decreasing dose [33, 41, 73, 74]
Acute kidney injury (AKI)	• No pre-clinical reports	• SIR-AE 3–5 AKIs after liver treatment (*n* = 9/146) [62] • AKIs after hepatic treatments (*n* = 6 patients) resolved with IV fluids or dialysis [8, 82, 101]	Nephropathy from large-volume tissue lysis; risk associated with overlapping treatment zones and large volume ablations [8, 62]
**Vascular adverse events**			
Hemorrhage	• Transient hematuria, petechiae, hematomas (including asymptomatic perinephric hematoma) [70] • No significant hemorrhage with supratherapeutic anticoagulation in porcine liver and kidney and canine prostate ablation [48, 97, 104]	• SIR-AE 3 (*n* = 3/139) and SIR-AE 5 in a patient with a MELD score of 27 (*n* = 1/139). Bleeding occurred with and without intraprocedural heparin [9] • HOPE#4Liver reported anemia, splenic hematoma, melena among major complications [50] • Abdominal-wall hematoma and melena, with a hemoglobin drop from 12.6 to 8.9 g/dL, requiring transfusion of 2-units [107].	Mechanism not established. Proposed theory is that cavitation overwhelms vessel wall so that histotripsy’s usual vessel-sparing fails. Excessive dosing, tumor neovasculature stability, cirrhosis, and heavily pre-treated liver favored to increase risks [9, 107]
Pseudoaneurysm (PSA)	• No pre-clinical reports, despite numerous vascular targeting studies	• Arterial PSA (*n* = 2); 9 mm segmental-artery PSA at 12 h and embolized POD2, and a 7 mm right-hepatic-artery PSA at day 15 and embolized day 16) [107] • Intratumoral and treatment-zone PSA (*n* = 3/139) [9]	Direct cavitation when treatment zone includes artery, causing acute arterial-wall injury. Detection ranged from immediate to about 2 weeks [9, 107]
Portal vein thrombosis (PVT)	• Non-occlusive, transient branch PVT in porcine hepatic treatments (*n* = 7/8, 4/5, 3/6, 9/10, and 4/15) [40, 42, 45, 48, 80]	• Multiple reports of PVT (*n* = 2, 1/8, 2/230, 1, 1/47) many managed post-operatively with therapeutic anticoagulation [13, 93, 101, 107, 116] • Anticoagulation: PVT (8/21) of central/near-central liver tumors mitigated by peri-procedural heparin [108]; one case extensive PVT with segmental infarct occurred despite intraprocedural heparin (5000 units) [107] • Hepatic hypoperfusion (*n* = 2/47) [116]	Direct cavitation when portal veins are included within the treatment zone, leading to endothelial injury and thrombus formation [45, 48]. Cavitation shear stress and turbulent flow drive platelet adhesion, activation, and aggregation; rationale for intraprocedural heparin [108]. Clusters at central or near-central lesions, lies adjacent to and extends from the treatment zone [93, 107, 108]
Hepatic vein thrombosis (HVT)	• Occlusive murine (*n* = 2/8) and non-occlusive porcine HVT (*n* = 3/6) [42, 45]	• No clinical reports	Direct cavitation when hepatic vein is included within the treatment zone, with thrombus propagating beyond the treatment focus [42, 45]
**General adverse events**			
Hyperprogression/accelerated progression	• No pre-clinical reports • One study showed statistically significant reduction in metastasis [64]	• One case of cholangiocarcinoma case of aggressive local and systemic progression after incomplete ablation and surgical resection [7]	Mechanism not established; attributed to a pro-inflammatory-cytokine response after sublethal or incomplete ablation [7]
Pain & post-procedural symptoms	• None to moderate discomfort across animal studies • Pain neutral-to-improved in canine osteosarcoma limb-sparing treatment [91]	• Treatment associated abdominal and back pain [50], some patients needing overnight observation and IV analgesia [31, 46, 93] • Treatment provided pain relief [56] • Urinary catheter pain, dysuria, and bladder spasm [75]	Mechanism not established; transient treatment-site or procedural pain [31, 46, 50] or attributed to urinary catheter rather than the ablation [75]
Infection/sepsis	• Prostatic abscess after prostate ablation (*n* = 1/8) [14] • Septic peritonitis after off-target small bowel ablation during porcine pancreatic ablation (*n* = 1/9) [21]	• Sepsis with recent endoscopic retrograde cholangiopancreatography [47] • Septic shock secondary to pneumonia [31]	Mechanism not established; reported infections were tied to other factors
Mortality	• No intraprocedural deaths reported • Early euthanasia (not spontaneous death) occurred in numerous studies and was reported inconsistently	• 4 total deaths reported within 30 days of treatment: 2 AKI [62], 1 hemorrhage/vascular injury [9], 1 respiratory failure with underlying compromise [44] • An Epic Cosmos cohort study found an 11.1% 30-day mortality after histotripsy treatment.*	Cause-specific, no shared mechanism

^*^Epic Cosmos cohort study was not included in the complete review given that it was published after the inclusion date; however, it is included here per expert recommendation

**Fig. 3:**
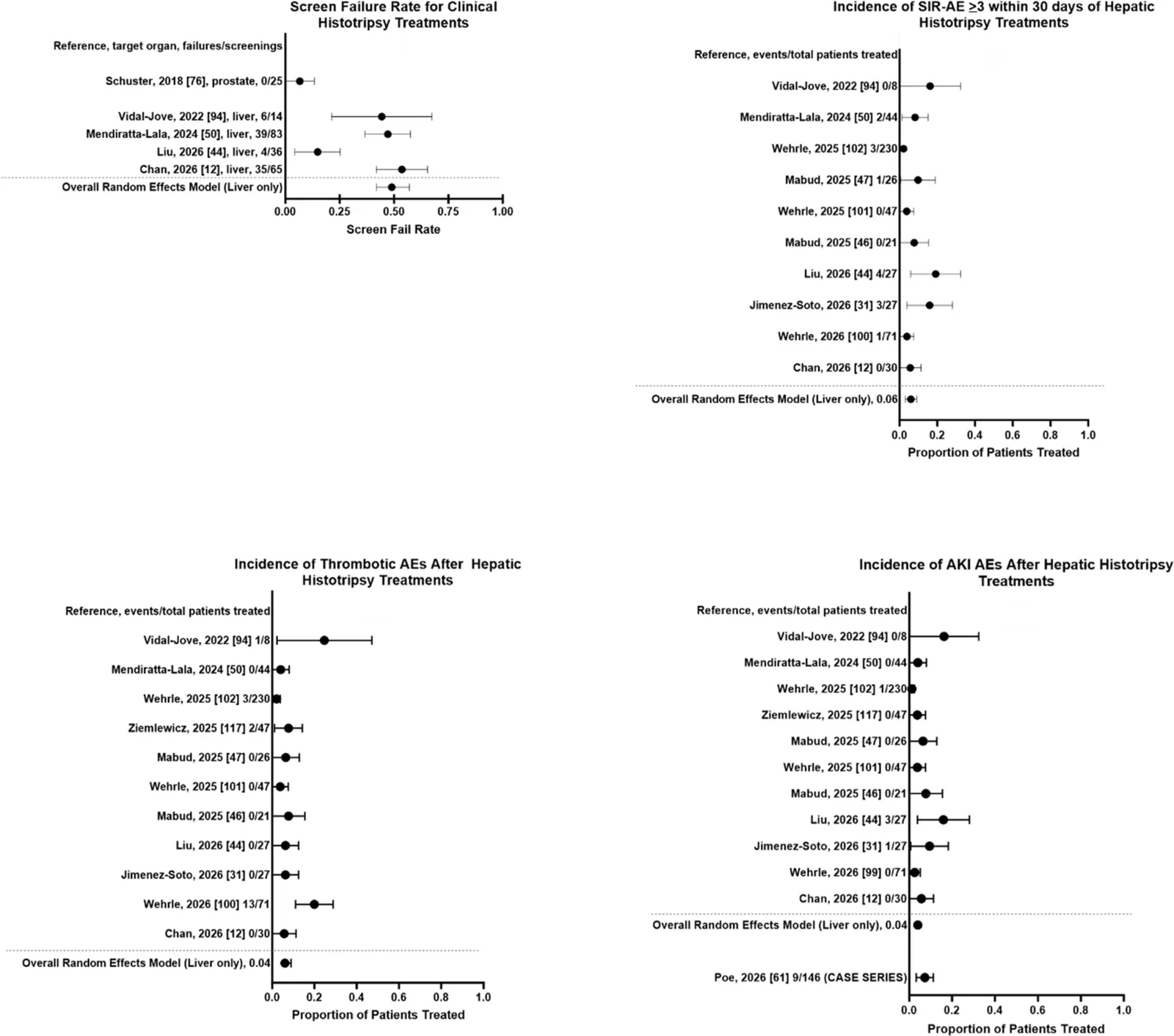
Forest plots for Histotripsy Screen Failure Rate and Incidence of SIR-AEs ≥ 3, Thrombotic AEs, and AKI AEs. Random effects models were used to determine pooled effects, with 95% confidence intervals graphed. Clinical cohort studies using Histotripsy for hepatic ablation were included for calculations. Screen failure from Schuster 2018 and AKIs from Poe 2026 were included in the figure for completeness and were not in the calculations

Meta-analysis of clinical cohort studies reviewed the most common events (Fig. 3). Screen-failure rate, patients who were deemed appropriate on clinical criteria, but who ultimately did not receive treatment, was 49% (95% CI: 47–52%). The overall incidence of SIR-AE 3 or greater was 6% (95% CI: 4–9%). The most common reported major AEs were thrombotic events and AKIs. Overall, AKIs had an incidence of 4% (95% CI: 2–6%). Thrombotic events had an incidence of 6% (95% CI: 4–9%).

### General injuries within the treatment zone and ultrasound path

Fifty-four studies reported injuries to non-target tissue prefocally or within the treatment zone. One study reported post-focal skin injuries.

#### Gastrointestinal system

Three bowel perforations (SIR-AE 3) occurred in a murine study using a custom 1 MHz transducer, high treatment parameters, and wide margins encompassing bowel [26]. Using the VORTX system, porcine pancreatic ablation reported similar, but mild bowel effects, without gross or histopathologic evidence of perforation [20].

Two SIR-AE 3 events were reported using the Edison system to target healthy porcine pancreatic tissue: septic peritonitis requiring euthanasia POD2 (postoperative day 2) and gastrointestinal obstruction [21]. Poor ultrasound visualization from overlying bowel gas was noted to be associated with bowel damage in 3/9 swine [21]. A later study treated pancreatic adenocarcinoma in swine using a custom 500 kHz transducer with altered pulsing schemes reported successful ablations without bowel injuries [30].

Two episodes of pancreatitis (SIR-AE 2) occurred in a study targeting healthy porcine pancreas, corresponding to ablation zones extending through the pancreatic capsule [21]. In the clinical treatment of a metastasis in hepatic segment 4b, acute interstitial pancreatitis developed post-treatment [107].

#### Skin and soft tissue

Nine studies reported minor pre-focal skin effects, not requiring intervention [9, 21, 36, 47, 60, 69, 99, 101, 116]. Clinically significant injury after treatments with the Edison included two skin injuries requiring topical ointment [100], skin blistering with admission for observation [46], and one body wall hematoma [107].

Two pre-clinical studies identified potential mechanisms of injury. In a canine soft-tissue sarcoma trial using a custom 500 kHz transducer on the VORTX system, residual hair potentiated pre-focal cavitation [66].

In the first limb-sparing use of Histotripsy for canine osteosarcoma, 4/24 treatments (16.7%) resulted in post-focal skin and soft-tissue injury, including two grade-3 ulcerations, attributed to post-focal thermal accumulation in the distal limb rather than pre-focal cavitation; reducing the pulse repetition frequency and pulses-per-point and adding a cooling pad lowered the event rate from 50% to 10% [91].

#### Pulmonary

In a porcine study using a 700 kHz pre-clinical prototype, 4/5 subjects had MR findings of right lower lobe lung injury following transcostal hepatic treatment, concordant with gross and histopathologic analysis [40]. One injury was moderate, with a sharply demarcated region, while the remaining three were mild. No pneumothoraxes were seen. One subject had no lung lesion on imaging or necropsy.

In a chronic rodent study, mice developed lower-lobe alveolar septal rupture with intra-alveolar pulmonary hemorrhage, attributed to the length of the acoustic channel relative to the small murine body; this injury was absent when the same protocol was applied to rats, underscoring the role of body size and acoustic-window geometry in beam-path lung injury [94].

#### Urinary system

Healthy canine prostates treated via a transabdominal transvesical approach with a custom 750 kHz system resulted in rectoprostatic fistulas (*n* = 2/21) and intraperitoneal uremia from prostatic urethral and capsular perforation (*n* = 2/21) [74]. A porcine survival model of Histotripsy targeting the proximal ureter and renal pelvis reported only low-grade, reversible urothelial injury on CT urography that resolved by day 7, with no bowel abnormalities in day-28 animals [37].

Distinct from these direct injuries to urinary-tract structures, acute kidney injury (AKI) following hepatic histotripsy has emerged as a systemic renal complication. In a five-center review of 146 procedures, 9 patients developed AKIs, graded SIR-AE 3 (*n* = 4), 4 (*n* = 2), and 5 (*n* = 2) [62]. Additional cases have been reported in case reports, MAUDE registry, and in a cohort report treating neuroendocrine liver metastases [8, 44, 101].

### Vascular injury and hemorrhage

Most bleeding-related adverse events were low-grade (SIR-AE 1–2) including serosal hemorrhage, ecchymosis, petechiae, clinically insignificant hematomas, and transient hematuria after hepatic, renal, and prostate treatment [98] (Table 4).

From a case report and a multicenter report, hepatic ablation using the Edison system required embolization and transfusions (*n* = 2; *n* = 4/139) for bleeding [9, 107]. Three patients required embolization, three had pseudoaneurysms within the treatment zone, and two developed hemorrhagic shock. One died on POD28 from fulminant hepatic failure [9].

Transient hematuria occurred in treatment of benign prostatic enlargement with the VORTX system, resolved by POD8 [75]. In the #HOPE4LIVER trial, post-procedural anemia was reported (*n* = 3/44), including one associated with a splenic hematoma and one with melena; No procedure-related hemorrhagic events or associated mortality occurred within 1-year (*n* = 47) [50, 116]. An early clinical study of histotripsy for renal cell carcinoma reported one case of post-procedure mild hematuria [98].

In a rat study of renal ablation, transient hematuria was accompanied by asymptomatic perinephric hematomas and localized petechial or subcapsular bleeding [70].

Pre-clinically, three anticoagulated large animal studies (*n* = 28 animals) did not find significant hemorrhage or hemoglobin decreases after histotripsy treatment of prostate, liver, and kidney [48, 97, 104].

### Thrombosis and embolization

Thrombosis and embolization were infrequently reported in early clinical trials [75, 93]. Subsequent multicenter studies demonstrated portal and hepatic vein thrombosis as one of the most common clinically significant AEs (SIR-AE 3–5) [50, 99, 101, 107]. A more recent investigation helped identify central tumor location and treatment overlap as important risk factors clinically [13, 108]. These findings aligned with the mechanistic work seen pre-clinically, which showed that thrombosis was associated with endothelial injury [40, 45, 48, 79].

Other pre-clinical studies evaluating Histotripsy in the kidneys reported ischemic changes, resolved by POD7 [33, 41].

### Metastases: disease progression versus abscopal effect

41 publications on Histotripsy malignant tumor ablation had at least one week of follow-up, including 18 clinical reports [7, 9, 10, 31, 46, 47, 50, 54, 55, 90, 92, 93, 98–101, 107, 116], 4 veterinary reports [3, 66, 67, 72], and 19 murine studies [4, 27–29, 49, 61, 63, 64, 70, 77, 78, 81, 83, 85, 86, 89, 109–111].

The majority of clinical (n = 11/18) and veterinary (n = 4/4) studies reported stable or progressive systemic disease acutely [3, 7, 9, 10, 21, 46, 50, 54, 55, 66, 67, 72, 90, 98, 100, 107, 116]. In five clinical reports, fourteen patients receiving variable systemic therapy demonstrated regression of untreated tumors on follow-up imaging, with some reporting concordant tumor marker decreases [31, 47, 54, 92, 99]. Notably, two pre-clinical studies suggest histotripsy may elicit statistically significant systemic tumor inhibition through immune-mediated mechanisms [49, 51].

One case of recurrent cholangiocarcinoma treated with Histotripsy subsequently developed hyperacute, extensive metastatic disease [7]. Immediate non-contrast imaging suggested complete treatment; however, regrowth was evident at 1 month. This mass was surgically excised 2 months after treatment with a diameter greater than double 1-month post-treatment imaging. 3 months after Histotripsy, approximately fifty metastatic lesions were found throughout the retroperitoneum and subcutaneous fat in an atypical pattern.

No animal studies showed an acute increase in systemic disease burden after histotripsy treatment when compared to control groups.

### Pain

Pain was infrequently and non-systematically reported. Large animal and clinical trials reported mild to moderate pain after ablation with no or minimal analgesic requirements [23, 31, 35, 50, 66, 75, 93, 99, 116]. One clinical patient treated for pancreatic cancer liver metastasis experienced significant post-procedural pain, requiring overnight observation [46]. In the canine osteosarcoma limb-sparing trial, pain was measured prospectively and was neutral-to-improved, with a significant increase in weight-bearing on the treated limb by the end of follow-up [91].

### Infection

Clinically, two patients’ liver ablations resulted in bacteremia that required antibiotic treatment, one of which had a compromised biliary system prior to ablation and required admission (SIR-AE 3) [47, 100].

### Mortality

Reported deaths were uncommon and cause-specific. Beyond the single death on POD28 from fulminant hepatic failure in the hepatic vascular-injury series [9], two deaths were attributed to grade-5 AKI [62], and one to respiratory failure in a patient with pre-existing respiratory compromise [44]. Pre-clinical mortality was limited to protocol-directed euthanasia (intraperitoneal urinoma; urine leak) and a single contrast-reaction death judged unrelated to the device. Although the study was not included in the review as it was published after the search date, a limited cohort study using data in Epic Cosmos found an 11.1% 30-day mortality rate [76]. The study did not further analyze the cause of death of these patients.

### Rare adverse events

Other AEs were from isolated reports. Clinically there are reports of hypertension with tachycardia [55], vomiting [98], pyrexia [101], congestive heart failure exacerbation [101], urinary retention [47], headache [31], and hyperglycemia [31]. Although not from a study included in this review given it did not have a parenchymal target, significant hemolysis was reported when treatments were endovascular [15].

## Discussion

Narrative reviews have summarized Histotripsy physics [6], techniques [105], immunologic effects [25], applications [18, 22, 115], and the emerging clinical experience [1, 102]. However, systematic evaluation of Histotripsy-associated AEs remains limited. This review is the first to synthesize pre-clinical and clinical AE evidence across parenchymal targets. Most studies reviewed here reported few AEs; however, some AEs were significant, including thromboses, vascular injuries, acute kidney injuries, and hyperprogression (Table 4).

Histotripsy risks damage to non-target anatomy within the treatment zone or from off-target cavitation. Pre-clinical bowel injuries highlight the consequences of poor visualization [21, 26], with additional pre-clinical studies stratifying the relative risk of injuries to luminal structures [38, 39]. Further, pre-clinical data show dose-dependent damage and varying effects with different pulsing schemes [28, 96, 112], whereas clinically there is a single treatment dose and pulsing scheme. Although speculative, this suggests that tissue selectivity may be further refined beyond current strategies.

Given the proximity of ablation targets to vasculature and the disorganized, thin-walled nature of tumor neovasculature, bleeding and vascular injury are important safety considerations. Traditional ablation carries a reported < 1% incidence of significant post-procedure hemorrhage, with delayed vascular injuries including pseudoaneurysms variably reported and managed conservatively or with embolization [5, 11, 16, 17, 34, 87, 88]. Post-Histotripsy bleeding was uncommon, reported with 4/139 patients with SIR-AE 3 vascular injury or hemorrhage under varied anticoagulation [9, 107]. Importantly, bleeding in renal and hepatic treatments was not worsened in three anticoagulated animal models [48, 97, 104]. Clinicians must remain vigilant for hemorrhage and stratify risk by known risk factors including tumor vascularity, anticoagulation, comorbidities (e.g., cirrhosis or coagulopathies), and ability to tolerate hypotension or resuscitation prior to embolization.

Mechanical disruption of tissue, vasculature, and platelets introduces a risk of thrombosis and embolization. Histotripsy debris particulates measure up to 54 µm [114], within the range of embolic agents, thus embolization is theoretically possible. Multiple pre-clinical reports of transient ischemic episodes resolved without intervention within 1 month [33, 41, 45, 48, 79]. These episodes were associated with thrombosis rather than debris embolization. The clinical incidence of thrombosis was 6% (95% CI: 4–8%, Fig. 3). One report described PVT with a segmental hepatic infarct after Histotripsy extending into the vein, despite prophylactic heparin, with partial recanalization by POD15 [107]. While the cause of thrombosis and management options have been discussed [108], future clinical studies should incorporate standardized post-procedure imaging protocols to prospectively evaluate for thrombotic and embolic events.

Tumor seeding and propagation are potential AEs when treating solid masses. Studies on other therapeutic ultrasound modalities report mixed findings, with some high-energy shock wave and cavitation-based studies demonstrating enhanced metastasis in highly metastatic models while others found a decrease [51–53, 58, 59, 113]. The theoretical risk is that viable cell clusters liberated during treatment could disseminate hematogenously. Hyperprogression or metastatic exacerbation following histotripsy was reported in a single clinical case report [7]. While causality cannot be established, this contrasts with pre-clinical and early clinical data suggesting potential immune-mediated systemic tumor suppression [25, 64, 92]. These findings highlight the need for mechanistic investigation and long-term oncologic surveillance to distinguish histotripsy effects from the natural history of aggressive disease and adjuvant therapies.

Several strategies may mitigate these AEs. Nontarget injury is best minimized through careful patient selection, pretreatment planning, a clear acoustic window, continuous bubble-cloud visualization, respiratory management, precise transducer alignment, and therapy pauses when needed. Planning should prioritize avoidance of susceptible structures, such as lungs and bowels, while maintaining visualization to verify treatment and detect unintended effects. Prophylactic anticoagulation, including low-dose heparin, mitigates thrombotic risk without hemorrhage in pre-clinical models. No routine prophylaxis is proven as safe for the vascular injury and hemorrhage recently identified clinically, which appear more common in areas of overlapping treatment zones. As clinical use expands beyond subcostal and inferior hepatic targets, continued reference to safety literature is essential.

Several limitations of the available evidence temper the conclusions of this review. Histotripsy data span fewer than 20 years, mostly from pre-clinical models with variable transducers and parameters; clinical data span only 6 years total, limited to single-arm trials, feasibility studies, case series, and case reports. Further, multiple publications are derived from overlapping cohorts, so the number of publications overrepresents the breadth of independent experience. The predominance of early-adopter centers and investigator-industry collaborations also introduces potential publication and reporting bias. Learning-curve effects may inflate the apparent frequency of AEs, while incomplete ascertainment (e.g., omitting labs needed to evaluate for AKIs) may lower it. Given the scarcity of detail on clinical system parameters, extrapolating pre-clinical and early clinical safety data to future treatments may be inappropriate. Exact clinical system therapeutic parameters need to be clearly defined, as required by the ablation standardization reporting lexicon [2]. Longer-term data on metastatic potential are also required, especially in slow-growing tumors that may not reveal occult micrometastases in the hyperacute setting.

Moving forward, open AE reporting among investigators is essential to ensure rigorous evaluation matches enthusiasm. Clinical adoption should remain grounded in risk awareness and rigorous science leading to high-level evidence.

## Data Availability

Not applicable.

## References

[1] Abi-Jaoudeh N, Ziemlewicz TJ, White S, Wah T, Cho C. Histotripsy for liver tumor ablation and beyond: AJR expert panel narrative review. Am J Roentgenol. 2026. 10.2214/AJR.26.34627. 42233891

[2] Ahmed M, Solbiati L, Brace CL, et al. Image-guided tumor ablation: standardization of terminology and reporting criteria—a 10-year update. Radiology. 2014;273(1):241–60. 10.1148/radiol.14132958 PMC4263618 24927329

[3] Ashar H, Singh A, Kishore D, et al. Enabling chemo-immunotherapy with HIFU in canine cancer patients. Ann Biomed Eng. 2024;52(7):1859–72. 10.1007/s10439-023-03194-1 PMC12323608 37162696

[4] Ashida R, Kawabata K-i, Maruoka T, et al. New approach for local cancer treatment using pulsed high-intensity focused ultrasound and phase-change nanodroplets. J Med Ultrason. 2015;42(4):457–66. 10.1007/s10396-015-0634-4 26576970

[5] Atwell TD, Carter RE, Schmit GD, et al. Complications following 573 percutaneous renal radiofrequency and cryoablation procedures. J Vasc Interv Radiol. 2012;23(1):48–54. 10.1016/j.jvir.2011.09.008 22037491

[6] Bader KB, Vlaisavljevich E, Maxwell AD. For whom the bubble grows: physical principles of bubble nucleation and dynamics in histotripsy ultrasound therapy. Ultrasound Med Biol. 2019;45:1056-80. 10.1016/j.ultrasmedbio.2018.10.035 PMC6524960 30922619

[7] Berman ZT, Patel T, Burgoyne AM, Clary BM. Hyperprogression and systemic metastasis of cholangiocarcinoma after histotripsy therapy. J Vasc Interv Radiol. 2025;36(9):1465–6. 10.1016/j.jvir.2025.06.002 40846437

[8] Bhutta S, Wanchoo R, Sakhiya V, Gholami S, Devoe C, Jhaveri KD. AKI after hepatic histotripsy. Kidney Int Rep. 2026;11(7):106603. 10.1016/j.ekir.2026.106603 PMC13254590 42292645

[9] Breuer JA, Mendiratta-Lala M, Lee Jr FT, et al. Vascular injury after histotripsy: a case series of hemorrhage and pseudoaneurysm complications in human patients. J Vasc Interv Radiol. 2026;37:108576. 10.1016/j.jvir.2026.108576 41655899

[10] Burns K, Juarez TM. Histotripsy for portal vein tumor thrombus in a patient with hepatocellular carcinoma: a case report. Front Oncol. 2025;15:1721814. 10.3389/fonc.2025.1721814 PMC12705397 41409237

[11] Ch’ng LS, Tazuddin EEM, Young B, Ali AFM. Spontaneous resolution of asymptomatic hepatic pseudoaneurysm post radiofrequency ablation. BJR| Case Rep. 2016;2(2):20150306. 10.1259/bjrcr.20150306 PMC6180864 30363592

[12] Chan AC, Lau VW, Wong GT, et al. Prospective analysis on the outcomes of histotripsy for primary and metastatic liver tumors. Int J Surg. 2026;112(2):3287–96. 10.1097/JS9.0000000000003856 41217432

[13] Cook M, Sears O, Sheeran D. Delayed hepatic and portal venous thrombosis following histotripsy: a single-institution case series. J Vasc Interv Radiol: JVIR. 2026;37:108864 10.1016/j.jvir.2026.108864 42184902

[14] Darnell SE, Hall TL, Tomlins SA, Cheng X, Ives KA, Roberts WW. Histotripsy of the prostate in a canine model: characterization of post-therapy inflammation and fibrosis. J Endourol. 2015;29(7):810–5. 10.1089/end.2014.0585 PMC4507352 25566880

[15] Devanagondi R, Zhang X, Xu Z, et al. Hemodynamic and hematologic effects of histotripsy of free-flowing blood: implications for ultrasound-mediated thrombolysis. J Vasc Interv Radiol. 2015;26(10):1559–65. 10.1016/j.jvir.2015.03.022 PMC4584157 25952642

[16] Doppelt G, Occéan B-V, Fabbro-Peray P, et al. The safety standards for liver percutaneous thermal ablation: a meta-analysis of adverse events over 15 years. Radiology. 2026;318(3):e251517. 10.1148/radiol.251517 41874301

[17] Facciorusso A, Abd El Aziz MA, Tartaglia N, et al. Microwave ablation versus radiofrequency ablation for treatment of hepatocellular carcinoma: a meta-analysis of randomized controlled trials. Cancers (Basel). 2020;12(12):3796. 10.3390/cancers12123796 PMC7766963 33339274

[18] Falk KL, Laeseke PF, Kisting MA, et al. Clinical translation of abdominal histotripsy: a review of preclinical studies in large animal models. Int J Hyperthermia. 2023;40(1):2272065. 10.1080/02656736.2023.2272065 PMC10629829 37875279

[19] Falk KL, Wagner MG, Hennen C, et al. Safety and efficacy of histotripsy treatments near implanted metallic fiducials and endovascular coils in phantoms and in an in vivo swine model. Ultrasound Med Biol. 2026;52:1736-44. 10.1016/j.ultrasmedbio.2026.04.025 PMC13334985 42185184

[20] Gannon J, Imran KM, Hendricks-Wenger A, et al. Ultrasound-guided noninvasive pancreas ablation using histotripsy: feasibility study in an in vivo porcine model. Int J Hyperth. 2023;40(1):2247187. 10.1080/02656736.2023.2247187 PMC10839746 37643768

[21] Gannon J, Paul T, Imran KM, et al. Non-invasive pancreas ablation using histotripsy: pre-clinical safety study in an in vivo porcine model. Ultrasound Med Biol. 2026;52:62-71. 10.1016/j.ultrasmedbio.2025.07.026 PMC12917764 41046199

[22] Hay AN, Ruger L, Hsueh A, et al. A review of the development of histotripsy for extremity tumor ablation with a canine comparative oncology model to inform human treatments. Int J Hyperth. 2023;40(1):2274802. 10.1080/02656736.2023.2274802 PMC10669778 37994796

[23] Hempel CR, Hall TL, Cain CA, Fowlkes JB, Xu Z, Roberts WW. Histotripsy fractionation of prostate tissue: local effects and systemic response in a canine model. J Urol. 2011;185(4):1484–9. 10.1016/j.juro.2010.11.044 PMC3075837 21334667

[24] Hendricks-Wenger A, Arnold L, Gannon J, et al. Histotripsy ablation in preclinical animal models of cancer and spontaneous tumors in veterinary patients: a review. IEEE Transact Ultrasonics Ferroelectrics Freq Contr. 2021;69(1):5-26. 10.1109/TUFFC.2021.3110083 PMC9284566 34478363

[25] Hendricks-Wenger A, Hutchison R, Vlaisavljevich E, Allen IC. Immunological effects of histotripsy for cancer therapy. Front Oncol. 2021;11:1999. 10.3389/fonc.2021.681629 PMC8200675 34136405

[26] Hendricks-Wenger A, Saunier S, Simon A, et al. Histotripsy for the treatment of cholangiocarcinoma in a patient-derived xenograft mouse model. Ultrasound Med Biol. 2022;48(2):293–303. 10.1016/j.ultrasmedbio.2021.10.002 34750030

[27] Hendricks-Wenger A, Sereno J, Gannon J, et al. Histotripsy ablation alters the tumor microenvironment and promotes immune system activation in a subcutaneous model of pancreatic cancer. IEEE Trans Ultrason Ferroelectr Freq Control. 2021;68(9):2987–3000. 10.1109/TUFFC.2021.3078094 PMC9295194 33956631

[28] Hendricks-Wenger A, Weber P, Simon A, et al. Histotripsy for the treatment of cholangiocarcinoma liver tumors: in vivo feasibility and ex vivo dosimetry study. IEEE Transact Ultrasonics Ferroelectrics Freq Contr. 2021;68:2953-64. 10.1109/TUFFC.2021.3073563 PMC9297335 33856990

[29] Hoogenboom M, Eikelenboom DC, van den Bijgaart RJ, et al. Impact of MR-guided boiling histotripsy in distinct murine tumor models. Ultrason Sonochem. 2017;38:1–8. 10.1016/j.ultsonch.2017.02.035 28633808

[30] Imran KM, Gannon J, Morrison HA, et al. Successful in situ targeting of pancreatic tumors in a novel orthotopic porcine model using histotripsy. Ultrasound Med Biol. 2023;49(11):2361–70. 10.1016/j.ultrasmedbio.2023.07.013 PMC10529075 37596154

[31] Jimenez-Soto C, Byrne MM, Loszko A, et al. First FDA approved experience with histotripsy for liver cancer management. Int J Surg. 202;112(3):7227-37. 10.1097/JS9.0000000000004070 41255274

[32] Juarez TM, Chuchua V, Burns K. Histotripsy for multifocal breast cancer liver metastases with early complete metabolic response: a case report. Front Oncol. 2026;16:1782397. 10.3389/fonc.2026.1782397 PMC13095606 42022317

[33] Khokhlova TD, Schade GR, Wang Y-N, et al. Pilot in vivo studies on transcutaneous boiling histotripsy in porcine liver and kidney. Sci Rep. 2019;9(1):20176. 10.1038/s41598-019-56658-7 PMC6934604 31882870

[34] Kim JW, Shin JH, Kim PN, et al. Embolization for bleeding after hepatic radiofrequency ablation. J Vasc Interv Radiol. 2017;28(3):356-365. e352. 10.1016/j.jvir.2016.09.031 28012690

[35] Kim Y, Fifer CG, Gelehrter SK, et al. Developmental impact and lesion maturation of histotripsy-mediated non-invasive tissue ablation in a fetal sheep model. Ultrasound Med Biol. 2013;39(6):1047–55. 10.1016/j.ultrasmedbio.2012.12.014 23453378

[36] Kim Y, Gelehrter S, Fifer C, et al. Non-invasive pulsed cavitational ultrasound for fetal tissue ablation: feasibility study in a fetal sheep model. Ultrasound Obstet Gynecol. 2011;37(4):450–7. 10.1002/uog.8880 21433165

[37] Kisting AL, Zlevor AM, Falk KL, et al. Histotripsy of the proximal ureter and renal pelvis: evaluation of urothelial injury in a porcine survival model. J Vasc Interv Radiol. 2025;36(3):512-520. e511. 10.1016/j.jvir.2024.11.028 PMC12067682 39662616

[38] Kisting MA, Kisting AL, Kurichety SL, et al. Histotripsy of the hepatic capsule and the gastric wall: acute treatment effects in a swine model. Int J Hyperth. 2025;42(1):2572364. 10.1080/02656736.2025.2572364 PMC12967312 41122018

[39] Kisting MA, White JK, Periyasamy S, et al. Safety and efficacy of histotripsy delivery through overlying gas-filled small bowel in an ex vivo swine model. Int J Hyperthermia. 2024;41(1):2369305. 10.1080/02656736.2024.2369305 PMC11224713 38897626

[40] Knott EA, Longo KC, Vlaisavljevich E, et al. Transcostal histotripsy ablation in an in vivo acute hepatic porcine model. Cardiovasc Intervent Radiol. 2021;44:1643–50. 10.1007/s00270-021-02914-1 34244841

[41] Knott EA, Swietlik JF, Longo KC, et al. Robotically-assisted sonic therapy for renal ablation in a live porcine model: initial preclinical results. J Vasc Interv Radiol. 2019;30(8):1293–302. 10.1016/j.jvir.2019.01.023 PMC6925588 31130365

[42] Knott EA, Zlevor AM, Hinshaw JL, et al. A comparison study of microwave ablation vs. histotripsy for focal liver treatments in a swine model. Eur Radiol. 2023;33(2):1050–62. 10.1007/s00330-022-09112-8 36048208

[43] Li H, Cai Z, He P, et al. Mechanical destruction of canine prostate using a novel low-intensity histotripsy based on a perfluoropentane-filled sodium alginate scaffold. Int J Hyperth. 2025;42(1):2579893. 10.1080/02656736.2025.2579893 41158084

[44] Liu E, von Breitenbuch P, Kemp A, et al. Histotripsy for neuroendocrine liver metastases: Early single-institution outcomes and safety. Surgery. 2026;194:110084. 10.1016/j.surg.2026.110084 41950812

[45] Longo KC, Knott EA, Watson RF, et al. Robotically assisted sonic therapy (RAST) for noninvasive hepatic ablation in a porcine model: mitigation of body wall damage with a modified pulse sequence. Cardiovasc Intervent Radiol. 2019;42(7):1016–23. 10.1007/s00270-019-02215-8 PMC7456499 31041527

[46] Mabud TS, Vergara M, Du J, et al. Histotripsy of pancreatic cancer liver metastases: early outcomes and imaging findings. World J Surg. 2025;49:1899-902. 10.1002/wjs.12646 40445073

[47] Mabud TS, Vergara M, Du J, et al. Histotripsy of liver metastases: short-term safety and imaging findings. CardioVasc Interv Radiol. 2025;48(11):1594-603. 10.1007/s00270-025-04183-8 41016946

[48] Mauch SC, Zlevor AM, Knott EA, et al. Hepatic and renal histotripsy in an anticoagulated porcine model. J Vasc Interv Radiol. 2023;34(3):386-394. e382. 10.1016/j.jvir.2022.11.034 PMC11223641 36503074

[49] McGinnis R, Song B, Kim H, et al. Histotripsy dose impacts treated tumor immune infiltration and survival outcomes in a murine B16F10 melanoma model. Cancers. 2025;17(23):3773. 10.3390/cancers17233773 PMC12691148 41374975

[50] Mendiratta-Lala M, Wiggermann P, Pech M, et al. The #HOPE4LIVER single-arm pivotal trial for histotripsy of primary and metastatic liver tumors. Radiology. 2024;312(3):e233051. 10.1148/radiol.233051 PMC11427859 39225612

[51] Miller DL, Dou C. Contrast-aided diagnostic ultrasound does not enhance lung metastasis in a mouse melanoma tumor model. J Ultrasound Med. 2005;24(3):349–54. 10.7863/jum.2005.24.3.349 15723847

[52] Miller DL, Dou C. The potential for enhancement of mouse melanoma metastasis by diagnostic and high-amplitude ultrasound. Ultrasound Med Biol. 2006;32(7):1097–101. 10.1016/j.ultrasmedbio.2006.03.013 16829323

[53] Miller DL, Dou C, Song J. Lithotripter shockwave-induced enhancement of mouse melanoma lung metastasis: dependence on cavitation nucleation. J Endourol. 2004;18(9):925–9. 10.1089/end.2004.18.925 15659934

[54] Ong ES, Wehrle CJ, Alassas MM. Distant tumor response in the pelvis after histotripsy of a metastatic sarcoma of the liver in a patient with differentiated liposarcoma. Am Surg. 2025;92(2):452-6. 10.1177/00031348251371287 40873348

[55] Ong ES, Wehrle CJ, Alassas MM. Use of histotripsy to rapidly relieve biliary obstruction in a patient with recurrent hilar cholangiocarcinoma. Am Surg. 2025;92(1):282-5. 10.1177/00031348251358442 40876872

[56] Ong ES, Wehrle CJ, Alassas MM. Distant tumor response in the pelvis after histotripsy of a metastatic sarcoma of the liver in a patient with differentiated liposarcoma. Am Surg. 2026;92(2):452–6. 10.1177/00031348251371287 40873348

[57] Ong ES, Wehrle CJ, Alassas MM. Use of histotripsy to rapidly relieve biliary obstruction in a patient with recurrent hilar cholangiocarcinoma. Am Surg. 2026;92(1):282–5. 10.1177/00031348251358442 40876872

[58] Oosterhof G, Cornel E, Smits G, Debruyne F, Schalken J. The influence of high-energy shock waves on the development of metastases. Ultrasound Med Biol. 1996;22(3):339–44. 10.1016/0301-5629(95)02051-9 8783466

[59] Oosterhof G, Cornel E, Smits G, Debruyne F, Schalken J. Influence of high-intensity focused ultrasound on the development of metastases. Eur Urol. 1997;32(1):91–5. 9266238

[60] Pahk KJ, Heo J, Joung C, Pahk K. Noninvasive mechanical destruction of liver tissue and tissue decellularisation by pressure-modulated shockwave histotripsy. Front Immunol. 2023;14:1150416. 10.3389/fimmu.2023.1150416 PMC10227506 37261363

[61] Pepple AL, Guy JL, McGinnis R, et al. Spatiotemporal local and abscopal cell death and immune responses to histotripsy focused ultrasound tumor ablation. Front Immunol. 2023;14:1012799. 10.3389/fimmu.2023.1012799 PMC9900174 36756111

[62] Poe LL, Kisting AL, Mendiratta-Lala M, et al. Histotripsy-induced acute kidney injury following liver tumor treatment: a preliminary report of 9 cases. J Vasc Interv Radiol. 2026;37(9):108882. 10.1016/j.jvir.2026.108882 42218940

[63] Qi T, Jing Y, Deng J, et al. Boiling histotripsy using dual-frequency protocol on murine breast tumor model and promotes immune activation. IEEE Transact Ultrasonics Ferroelectrics Freq Contr. 2023;70:1776-85. 10.1109/TUFFC.2023.3326561 37871099

[64] Qu S, Worlikar T, Felsted AE, et al. Non-thermal histotripsy tumor ablation promotes abscopal immune responses that enhance cancer immunotherapy. J Immunother Cancer. 2020;8(1):1–12. 10.1136/jitc-2019-000200 PMC7057529 31940590

[65] Ruger L, Yang E, Coutermarsh-Ott S, et al. Histotripsy ablation for the treatment of feline injection site sarcomas: a first-in-cat in vivo feasibility study. Int J Hyperth. 2023;40(1):2210272. 10.1080/02656736.2023.2210272 PMC10278555 37196996

[66] Ruger L, Yang E, Gannon J, et al. Mechanical high-intensity focused ultrasound (histotripsy) in dogs with spontaneously occurring soft tissue sarcomas. IEEE Trans Biomed Eng. 2022;70(3):768–79. 10.1109/TBME.2022.3201709 PMC9969335 36006886

[67] Ruger LN, Hay AN, Gannon JM, et al. Histotripsy ablation of spontaneously occurring canine bone tumors. IEEE Trans Biomed Eng. 2022;70(1):331–42. 10.1109/TBME.2022.3191069 PMC9921194 35834467

[68] Ruger LN, Hay AN, Gannon JM, et al. Histotripsy ablation of spontaneously occurring canine bone tumors. 2023. 10.1109/TBME.2022.3191069 PMC9921194 35834467

[69] Ruger LN, Hay AN, Vickers ER, et al. Characterizing the ablative effects of histotripsy for osteosarcoma: in vivo study in dogs. Cancers. 2023;15(3):741. 10.3390/cancers15030741 PMC9913343 36765700

[70] Schade G, Wang Y, D’Andrea S, Hwang J, Liles W, Khokhlova T. Boiling histotripsy ablation of renal cell carcinoma in the eker rat promotes a systemic inflammatory response. Ultrasound Med Biol. 2019;45(1):137–47. 10.1016/j.ultrasmedbio.2018.09.006 PMC6546431 30340920

[71] Schade GR, Hall TL, Roberts WW. Urethral-sparing histotripsy of the prostate in a canine model. Urology. 2012;80(3):730–5. 10.1016/j.urology.2012.05.027 PMC3429725 22840869

[72] Schade GR, Keller J, Ives K, et al. Histotripsy focal ablation of implanted prostate tumor in an ACE-1 canine cancer model. J Urol. 2012;188(5):1957–64. 10.1016/j.juro.2012.07.006 PMC4296596 22999534

[73] Schade GR, Styn NR, Hall TL, Roberts WW. Endoscopic assessment and prediction of prostate urethral disintegration after histotripsy treatment in a canine model. J Endourol. 2012;26(2):183–9. 10.1089/end.2011.0349 PMC3311909 22050511

[74] Schade GR, Styn NR, Ives KA, Hall TL, Roberts WW. Prostate histotripsy: evaluation of prostatic urethral treatment parameters in a canine model. BJU Int. 2014;113(3):498–503. 10.1111/bju.12333 PMC3944657 24176120

[75] Schuster TG, Wei JT, Hendlin K, Jahnke R, Roberts WW. Histotripsy treatment of benign prostatic enlargement using the Vortx Rx system: initial human safety and efficacy outcomes. Urology. 2018;114:184–7. 10.1016/j.urology.2017.12.033 29330000

[76] Sears O, Bitar ER, Cummins KC, et al. Early outcomes of histotripsy for liver tumors in US clinical practice. JAMA Netw Open. 2026;9(8):e2627493. 10.1001/jamanetworkopen.2026.27493 PMC13444801 42555042

[77] Singh A, Chandrasekar SV, Valappil VT, Scaria J, Ranjan A. Tumor immunomodulation by nanoparticle and focused ultrasound alters gut microbiome in a sexually dimorphic manner. Theranostics. 2025;15(1):216. 10.7150/thno.99664 PMC11667224 39744230

[78] Singh MP, Sethuraman SN, Miller C, Malayer J, Ranjan A. Boiling histotripsy and in-situ CD40 stimulation improve the checkpoint blockade therapy of poorly immunogenic tumors. Theranostics. 2021;11(2):540. 10.7150/thno.49517 PMC7738858 33391491

[79] Smolock ARCM, Vlaisavljevich E, Gendron-Fitzpatrick A, Green C, Ziemlewicz TJ, Lee FT. Robotically assisted sonic therapy as a non-invasive non-thermal ablation modality: proof of concept in a porcine liver model. Radiology. 2018;287(2):485–93. 10.1148/radiol.2018171544 29381870

[80] Smolock AR, Cristescu MM, Vlaisavljevich E, et al. Robotically assisted sonic therapy as a noninvasive nonthermal ablation modality: proof of concept in a porcine liver model. Radiology. 2018;287(2):485–93. 10.1148/radiol.2018171544 29381870

[81] Song B, Queen H, Ferris SF, et al. Histotripsy-focused ultrasound treatment abrogates tumor hypoxia responses and stimulates antitumor immune responses in melanoma. Mol Cancer Ther. 2025;24(7):1088–98. 10.1158/1535-7163.MCT-24-0715 PMC12221799 40105625

[82] Staley C, Dakkak T, Robinson SS. Royall NA (2026) Observed immune response following histotripsy in metastatic hepatocellular carcinoma. Case Rep Oncol Med. 2026;1:4245816. 10.1155/crom/4245816 PMC13090675 42004160

[83] Stepanechko M, Gamache A, Padilla F, et al. Single treatment boiling histotripsy focused ultrasound ablation neither negates nor enhances the activity of α-CD40 in a pancreatic cancer model. IEEE Transact Biomed Eng. 2025;72(11):3404-18. 10.1109/TBME.2025.3568594 PMC12779120 40372859

[84] Styn N, Hall TL, Fowlkes JB, Cain CA, Roberts WW. Histotripsy homogenization of the prostate: thresholds for cavitation damage of periprostatic structures. J Endourol. 2011;25(9):1531–5. 10.1089/end.2010.0648 PMC3168978 21815807

[85] Styn NR, Hall TL, Fowlkes JB, Cain CA, Roberts WW. Histotripsy of renal implanted VX-2 tumor in a rabbit model: investigation of metastases. Urology. 2012;80(3):724–9. 10.1016/j.urology.2012.06.020 22925247

[86] Styn NR, Wheat JC, Hall TL, Roberts WW. Histotripsy of VX-2 tumor implanted in a renal rabbit model. J Endourol. 2010;24(7):1145–50. 10.1089/end.2010.0123 20575696

[87] Takaki H, Yamakado K, Nakatsuka A, et al. Frequency of and risk factors for complications after liver radiofrequency ablation under CT fluoroscopic guidance in 1500 sessions: single-center experience. AJR Am J Roentgenol. 2013;200(3):658–64. 10.2214/AJR.12.8691 23436859

[88] Tashi S, Gogna A, Leong S, Venkatanarasimha N, Chandramohan S. Vascular complications related to image-guided percutaneous thermal ablation of hepatic tumors. Diagn Interv Radiol. 2023;29(2):318. 10.5152/dir.2022.21809 PMC10679696 36987983

[89] Thim EA, Kitelinger LE, Rivera-Escalera F, et al. Focused ultrasound ablation of melanoma with boiling histotripsy yields abscopal tumor control and antigen-dependent dendritic cell activation. Theranostics. 2024;14(4):1647. 10.7150/thno.92089 PMC10879863 38389838

[90] Uysal M, Wehrle CJ, Coppa C, et al. Bridging therapy with histotripsy prior to liver transplantation for hepatocellular carcinoma: a first case report. Exp Hematol Oncol. 2025;14(1):20. 10.1186/s40164-025-00604-z PMC11863397 40001149

[91] Vickers ER, Ruger LN, Hay AN, et al. The first limb-sparing use of histotripsy for canine osteosarcoma. Sci Rep. 2026;16(1):14574. 10.1038/s41598-026-42319-z PMC13153271 41872305

[92] Vidal-Jové J, Serres-Créixams X, Ziemlewicz T, Cannata JM (2021) Liver histotripsy mediated abscopal effect. Case Report. IEEE Transact Ultrasonics Ferroelectr Freq Control. 2021;68(9):3001-5. 10.1109/TUFFC.2021.3100267 34310299

[93] Vidal-Jove J, Serres X, Vlaisavljevich E, et al. First-in-man histotripsy of hepatic tumors: the THERESA trial, a feasibility study. Int J Hyperthermia. 2022;39(1):1115–23. 10.1080/02656736.2022.2112309 36002243

[94] Vlaisavljevich E, Greve J, Cheng X, et al. Non-invasive ultrasound liver ablation using histotripsy: chronic study in an in vivo rodent model. Ultrasound Med Biol. 2016;42(8):1890–902. 10.1016/j.ultrasmedbio.2016.03.018 PMC4912895 27140521

[95] Vlaisavljevich E, Kim Y, Owens G, Roberts W, Cain C, Xu Z. Effects of tissue mechanical properties on susceptibility to histotripsy-induced tissue damage. Phys Med Biol. 2013;59(2):253. 10.1088/0031-9155/59/2/253 PMC4888779 24351722

[96] Vlaisavljevich E, Kim Y, Owens G, Roberts W, Cain C, Xu Z. Effects of tissue mechanical properties on susceptibility to histotripsy-induced tissue damage. Phys Med Biol. 2014;59(2):253–70. 10.1088/0031-9155/59/2/253 PMC4888779 24351722

[97] Vlaisavljevich E, Owens G, Lundt J, et al. Non-invasive liver ablation using histotripsy: preclinical safety study in an in vivo porcine model. Ultrasound Med Biol. 2017;43(6):1237–51. 10.1016/j.ultrasmedbio.2017.01.016 28318889

[98] Wah TM, Abdel-Hadi O, Brandon J, et al. Early clinical histotripsy treatment of renal cell carcinoma. Cardiovasc Intervent Radiol. 2025;48(6):883–5. 10.1007/s00270-025-04023-9 PMC12170758 40295402

[99] Wehrle CJ, Ahmed AFS, Knott E, et al. The first full year of histotripsy for liver tumors: Local tumor control and preliminary oncologic efficacy. Surgery. 2025;91:109898. 10.1016/j.surg.2025.109898 41353073

[100] Wehrle CJ, Burns K, Mabud T, et al. Local tumor control of liver tumors after histotripsy: a preliminary national multicenter study. JCO Oncol Pract. 2025:OP–25–00550. 10.1200/OP-25-00550 41401400

[101] Wehrle CJ, Burns K, Ong E, et al. The first international experience with histotripsy: a safety analysis of 230 cases. J Gastrointest Surg. 2025;29(4):102000. 10.1016/j.gassur.2025.102000 39978577

[102] Wehrle CJ, Lee J, Ahmed AS, et al. Histotripsy for liver tumours: a systematic review and meta-analysis of current clinical evidence. Eclinicalmedicine. 2026;95:103926. 10.1016/j.eclinm.2026.103926 PMC13139980 42094235

[103] Wei Y, Li S, Wang Q, et al. Non-invasive ultrasound liver ablation using histotripsy: a feasibility study in ex vivo porcine liver and a chronic study in rodent models in vivo. Ultrasound Med Biol. 2026;52(8):1483-91. 10.1016/j.ultrasmedbio.2026.03.001 42091361

[104] Wheat JC, Hall TL, Hempel CR, Cain CA, Xu Z, Roberts WW. Prostate histotripsy in an anticoagulated model. Urology. 2010;75(1):207–11. 10.1016/j.urology.2009.09.021 PMC2813892 19931897

[105] Williams RP, Simon JC, Khokhlova VA, Sapozhnikov OA, Khokhlova TD. The histotripsy spectrum: differences and similarities in techniques and instrumentation. Int J Hyperth. 2023;40(1):2233720. 10.1080/02656736.2023.2233720 PMC10479943 37460101

[106] Winterroth F, Xu Z, Wang T-Y, et al. Examining and analyzing subcellular morphology of renal tissue treated by histotripsy. Ultrasound Med Biol. 2011;37(1):78–86. 10.1016/j.ultrasmedbio.2010.10.002 PMC3038584 21144960

[107] Wong BO, Fan C-M, Shyn PB, Wang J. Vascular complications following histotripsy of hepatocellular carcinoma. Int J Hyperthermia. 2025;42(1):2603108. 10.1080/02656736.2025.2603108 41403239

[108] Worlikar T, Caoili E, Navarro A, et al. Management and prevention of histotripsy-induced acute bland portal vein thrombosis in histotripsy treated liver tumors. J Vasc Interv Radiol. 2026;37:108883 10.1016/j.jvir.2026.108883 42235819

[109] Worlikar T, Mendiratta-Lala M, Vlaisavljevich E, et al. Effects of histotripsy on local tumor progression in an in vivo orthotopic rodent liver tumor model. BME frontiers. 2020;2020:9830304. 10.34133/2020/9830304 PMC8318009 34327513

[110] Worlikar T, Vlaisavljevich E, Gerhardson T, et al. Histotripsy for non-invasive ablation of hepatocellular carcinoma (HCC) tumor in a subcutaneous xenograft murine model. Conf Proc IEEE Eng Med Biol Soc. 2018;2018:6064–7. 10.1109/EMBC.2018.8513650 30441719

[111] Worlikar T, Zhang M, Ganguly A, et al. Impact of histotripsy on development of intrahepatic metastases in a rodent liver tumor model. Cancers (Basel). 2022;14(7):1612. 10.3390/cancers14071612 PMC8996987 35406383

[112] Worlikar T, Zhang M, Yu M, et al. Histotripsy dose impacts tumor cellular damage and treatment outcomes in a preclinical model of hepatocellular carcinoma. Sci Rep. 2026;16(1):19156. 10.1038/s41598-026-48331-7 PMC13280371 42034785

[113] Wu F, Wang Z-B, Jin C-B, et al. Circulating tumor cells in patients with solid malignancy treated by high-intensity focused ultrasound. Ultrasound Med Biol. 2004;30(4):511–7. 10.1016/j.ultrasmedbio.2003.12.004 15121253

[114] Xu Z, Fan Z, Hall TL, Winterroth F, Fowlkes JB, Cain CA. Size measurement of tissue debris particles generated from pulsed ultrasound cavitational therapy–histotripsy. Ultrasound Med Biol. 2009;35(2):245–55. 10.1016/j.ultrasmedbio.2008.09.002 PMC2706707 19027218

[115] Xu Z, Khokhlova TD, Cho CS, Khokhlova VA. Histotripsy: a method for mechanical tissue ablation with ultrasound. Ann Rev Biomed Eng. 2024;26(1):141-67. 10.1146/annurev-bioeng-073123-022334 PMC11837764 38346277

[116] Ziemlewicz TJ, Critchfield JJ, Mendiratta-Lala M, et al. The #HOPE4LIVER single-arm pivotal trial for histotripsy of primary and metastatic liver tumors: 1-year update of clinical outcomes. Ann Surg. 2025;282(6):908-16. 10.1097/SLA.0000000000006720 PMC12594125 40201962

